# Treatment of Small Peri‐Implant Buccal Dehiscence Defects With SCTG or GBR: 3‐Year Results of a Pilot Study

**DOI:** 10.1111/cid.70192

**Published:** 2026-10-29

**Authors:** Anina N. Zuercher, Fabienne Rusch, Franz J. Strauss, Ronald E. Jung, Hyun‐Chang Lim, Daniel Thoma

**Affiliations:** ^1^ Clinic of Reconstructive Dentistry, Center of Dental Medicine University of Zurich Zurich Switzerland; ^2^ Discipline of Periodontics, The University of Sydney, School of Dentistry Sydney New South Wales Australia; ^3^ Universidad Autonoma de Chile Santiago Chile; ^4^ Department of Periodontology, Periodontal‐Implant Clinical Research Institute Kyung Hee University College of Dentistry, Kyung Hee University Medical Center Seoul Korea; ^5^ Department of Periodontology, Research Institute for Periodontal Regeneration Yonsei University College of Dentistry Seoul South Korea

**Keywords:** dental implants, guided bone regeneration, peri‐implant health, small buccal bone dehiscence, soft tissue augmentation

## Abstract

**Objective:**

To evaluate and compare subepithelial connective tissue grafts (SCTG) and guided bone regeneration (GBR) for the treatment of small peri‐implant buccal bone dehiscences over a 3‐year follow‐up, in terms of profilometric contour change, peri‐implant parameters and patient‐reported outcome measures (PROMs).

**Material and Methods:**

Patients presenting with small buccal bone dehiscences (≤ 3 mm) after single implant placement were included. Defect sites were randomly treated with either SCTG or GBR, while five patients without dehiscences served as a negative control group. All patients received an implant crown and were followed for 3 years. Profilometric measurements, marginal bone levels, peri‐implant parameters, and PROMs were recorded at baseline, 1, and 3 years. Data were analyzed descriptively.

**Results:**

A total of 16 patients were available for the 3‐year follow‐up (SCTG, *n* = 5; GBR, *n* = 6; negative control, *n* = 5). Both augmentation approaches maintained stable buccal soft‐tissue contours. At 1 mm below the mucosal margin, the median buccal contour change was 0.19 mm (Q1: −0.28; Q3: 0.29) in the SCTG group and 0.11 mm (Q1: −0.20; Q3: 0.30) in the GBR group. At 3 mm below the mucosal margin, the corresponding median contour changes were 0.05 mm (Q1: −0.36; Q3: 0.31) for SCTG and −0.05 mm (Q1: −0.22; Q3: 0.07) for GBR. Peri‐implant clinical parameters (probing depth, bleeding on probing, and plaque) remained stable, indicating maintained peri‐implant health in both groups. Marginal bone levels were preserved throughout the observation period and oral health‐related quality of life remained high and comparable between the SCTG and GBR groups.

**Conclusion:**

Within the limitations of this pilot study, both SCTG and GBR were associated with stable buccal contour conditions and favorable peri‐implant health over a 3‐year follow‐up period. These findings were observed under adequate self‐performed plaque control and adherence to supportive care. Adequately powered RCTs are needed to confirm these findings.

## Graphical abstract

This pilot randomized controlled trial evaluated SCTG and GBR for small peri‐implant buccal dehiscence defects (≤ 3 mm). At 3 years, both approaches showed stable buccal contours, favorable peri‐implant health and low patient‐reported oral health impact under adequate plaque control and supportive care. Larger, adequately powered trials are needed to confirm these findings.

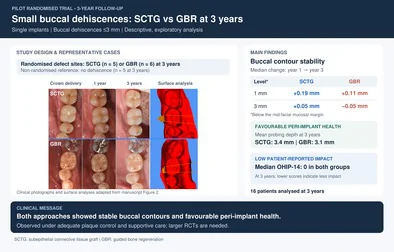

## 1 Introduction

The long‐term success of dental implants depends not only on predictable osseointegration but also on the maintenance of healthy and stable peri‐implant tissues [1]. Once integrated, the peri‐implant mucosa forms a protective soft‐tissue barrier that shields the underlying bone from microbial and mechanical challenges [2, 3]. The integrity of this mucosal barrier is therefore essential for long‐term implant success and aesthetic stability [4, 5, 6].

In many clinical situations, particularly after tooth extraction, small buccal bone dehiscences (≤ 3 mm) are present at implant placement. These defects represent a frequent and clinically relevant challenge, as they may compromise buccal contour stability and soft‐tissue support over time [7, 8]. A reduced buccal bone dimension is often associated with a thinner peri‐implant mucosa, which may predispose the site to contour flattening, mucosal recession and increased susceptibility to marginal bone remodeling [8, 9, 10]. Consequently, various augmentation strategies have been proposed to manage these small defects, aiming to preserve the natural ridge convexity and to establish a thick and stable peri‐implant mucosa [11, 12, 13].

Among the available augmentation techniques, guided bone regeneration (GBR) remains the gold standard for managing peri‐implant dehiscence‐type defects [14, 15, 16]. However, subepithelial connective‐tissue grafts (SCTG) have increasingly been applied as a biologically driven alternative, particularly in cases with small buccal dehiscences where soft‐tissue support is critical [12, 13]. From a biological perspective, the peri‐implant mucosa forms a continuous epithelial and connective tissue interface surrounding the implant, thereby sealing the transmucosal region from the oral environment [5, 17]. This soft‐tissue complex acts as a biological barrier that limits bacterial penetration and helps reduce inflammatory reactions [5, 18]. Deficiencies in the supporting buccal bone, however, may compromise the stability of this soft‐tissue seal and negatively affect long‐term contour stability and aesthetic outcomes.

While GBR primarily aims at hard‐tissue regeneration and re‐establishment of the buccal bone contour, the newly formed bone may undergo partial remodeling and resorption over time, which can limit its long‐term contribution to soft‐tissue stability [15, 19]. In contrast, SCTG primarily aims to increase mucosal thickness and improve soft‐tissue quality, thereby reinforcing the peri‐implant seal and supporting contour stability even in the absence of complete bone regeneration [20].

In a recent randomized controlled trial (RCT) comparing SCTG and GBR for the treatment of small buccal dehiscence defects, both approaches resulted in favorable clinical outcomes after 1 year, with SCTG demonstrating greater soft‐tissue volume gain and similar peri‐implant health parameters. However, the study was limited to short‐term observations and did not allow conclusions regarding the stability of these outcomes over time. In particular, it remains unclear whether the initial advantages observed with soft‐tissue augmentation are maintained in the mid‐term, or whether tissue remodeling and dimensional changes may alter the clinical outcomes beyond the first year [12].

Therefore, evidence regarding mid‐term stability of peri‐implant soft tissue contour and tissue dimensions following different augmentation approaches in small buccal dehiscence defects remains limited. Clarifying the long‐term behavior of these tissues is essential to better understand the biological and clinical implications of selecting hard versus soft‐tissue augmentation strategies at the time of implant placement.

Following these 1‐year results [12], the present 3‐year analysis aimed to evaluate the peri‐implant tissue conditions and stability of the two augmentation modalities, SCTG and GBR, for the treatment of small buccal dehiscences (≤ 3 mm) at implant placement. The study specifically assessed their mid‐term performance in terms of profilometric, clinical, and patient‐reported outcome measures (PROMs).

## 2 Materials and Methods

### 2.1 Study Design

The present study represents the 3‐year follow‐up of a previously published RCT that compared subepithelial connective tissue grafts (SCTG) and guided bone regeneration (GBR) for the treatment of small buccal peri‐implant dehiscence defects [12]. The original study was designed as a pilot RCT with an exploratory character due to the limited sample size. The 3‐year follow‐up was conducted as a continuation of the original study protocol and approved accordingly. It was performed at the Clinic of Reconstructive Dentistry, Center of Dental Medicine, University of Zurich, Switzerland.

The randomization in the original trial was performed using a computer‐generated random allocation sequence. Allocation concealment was ensured by using sealed opaque envelopes, which were opened at the time of surgery after confirmation of the presence of a buccal bone dehiscence defect. The allocation was implemented by a study coordinator not involved in outcome assessment. Randomization was applied only to sites presenting a buccal bone dehiscence defect (≤ 3 mm), which were allocated to either SCTG or GBR. In addition, a group of patients without buccal bone defects at implant placement was included as a non‐randomized reference cohort.

The present follow‐up analysis evaluates profilometric contour changes, peri‐implant parameters, and patient‐reported outcome measures (PROMs) to assess the mid‐term outcomes of the two augmentation approaches. The original trial was registered at the German Clinical Trials Register (DRKS00012733) and approved by the local ethics committee (BASEC‐ID 2017‐01401). The present 3‐year follow‐up was conducted as an extension of the original study protocol and approved by the local ethics committee. The reporting of the study follows the CONSORT guidelines.

### 2.2 Study Population

A detailed description of the inclusion and exclusion criteria was previously provided [12]. In brief, patients aged ≥ 18 years were included if they required single‐tooth implant placement in the posterior maxilla or mandible and presented good general and periodontal health. Eligible participants showed full‐mouth plaque and bleeding scores below 25%, probing depths < 4 mm, and at least one adjacent tooth and an opposing dentition. Patients were excluded if they presented systemic diseases that could interfere with bone or soft‐tissue healing, were pregnant or breastfeeding, had active periodontal disease, smoked more than 15 cigarettes per day, or showed poor oral hygiene or compliance. All patients provided written informed consent prior to inclusion and were treated according to the same surgical and prosthetic protocol. For the present study, only patients who completed the 3‐year follow‐up examination were included in the final analysis.

### 2.3 Clinical Procedures

The surgical procedure has been described in detail in the previous publication [12]. In short, at the surgical site, the implant bed was prepared according to the manufacturer's recommendations and a titanium dental implant (OsseoSpeed EV, Dentsply Sirona Implants, Mölndal, Sweden; diameter: 3.6 mm for premolar sites and 4.2 mm for molar sites) was placed in a prosthetically driven position. At the time of implant placement, following flap elevation and implant insertion, the presence and size of the buccal bone dehiscence defect were assessed intraoperatively. The vertical extent of the defect was measured from the implant shoulder to the most coronal point of the buccal bone using a periodontal probe (PCPUNC 15, Hu‐Friedy, Chicago, IL, USA). Measurements were performed under direct visualization by the operating surgeon. Defects ≤ 3 mm were considered eligible for inclusion and subsequent randomization. Thereafter, small buccal bone dehiscence defects (≤ 3 mm) were randomly allocated to one of two treatment modalities: 1. subepithelial connective tissue graft (SCTG) or 2. guided bone regeneration (GBR). To provide a reference for physiological healing conditions, an additional group of patients without buccal bone defects at implant placement was included as a non‐randomized reference cohort. In all study groups, a healing abutment was connected and a transmucosal healing approach was applied. After a healing period of 3–6 months, the prosthetic phase was initiated. A titanium abutment (TitaniumBase EV, Dentsply Sirona Implants) and a monolithic multilayered zirconia crown were fabricated following a conventional impression technique. The restorations were individually fabricated by different dental laboratories and customized in shade to achieve harmonious integration with the adjacent dentition. All crowns were screw‐retained, with the screw access channel sealed with Teflon tape and a composite resin filling and were delivered to the patients. The patients were thereafter followed up for 3 years.

### 2.4 Baseline and Clinical Follow‐Up Examinations

The baseline examination (crown delivery) was performed 7–10 days after delivery of the final restoration, followed by follow‐up examinations at 1 and 3 years after implant loading. During these visits, clinical and peri‐implant parameters—including plaque control records (PCR), probing depth (PD), bleeding on probing (BOP), and width of keratinized mucosa (KM)—were recorded. In addition, digital impressions, cone‐beam computed tomographic (CBCT) scans, and standardized periapical radiographs were obtained, and patients completed the Oral Health Impact Profile (OHIP‐14) questionnaire to assess patient‐reported outcomes.

### 2.5 Outcome Measures

#### 2.5.1 Primary Outcome Measure: Buccal Contour Changes

For the evaluation of volumetric soft tissue contour changes, conventional impressions were taken at crown delivery, 1 and 3 years. STL surface scans were obtained by optical scanning of the conventional impressions. The STL files from the different time points were superimposed and aligned to adjacent tooth surfaces using a best‐fit algorithm (SMOP software, Swissmeda AG, Zurich, Switzerland).

For the profilometric measurements, a standardized region of interest (ROI) was defined at the mid‐facial aspect of each implant crown. The ROI boundaries were set as follows: 1 mm apical to the mid‐facial mucosal margin (coronal boundary), the mucogingival junction (apical boundary), and 1 mm from the adjacent tooth surfaces (mesial and distal boundaries). Within this ROI, volumetric and linear changes were quantified at 1 and 3 mm below the mucosal margin. Profilometric measurements were expressed in mm as the mean distance change between surface datasets obtained at baseline, 1, and 3 years.

For the linear measurements, a bucco‐oral cross‐section through the central implant axis was used to determine the horizontal distance between the different time points. The tissue width (TW) was measured horizontally at 1 mm (TW‐1) and 3 mm (TW‐3) below the mid‐facial mucosal margin (Figure 1). These measurements represented the changes in peri‐implant tissue width over time.

**FIGURE 1:**
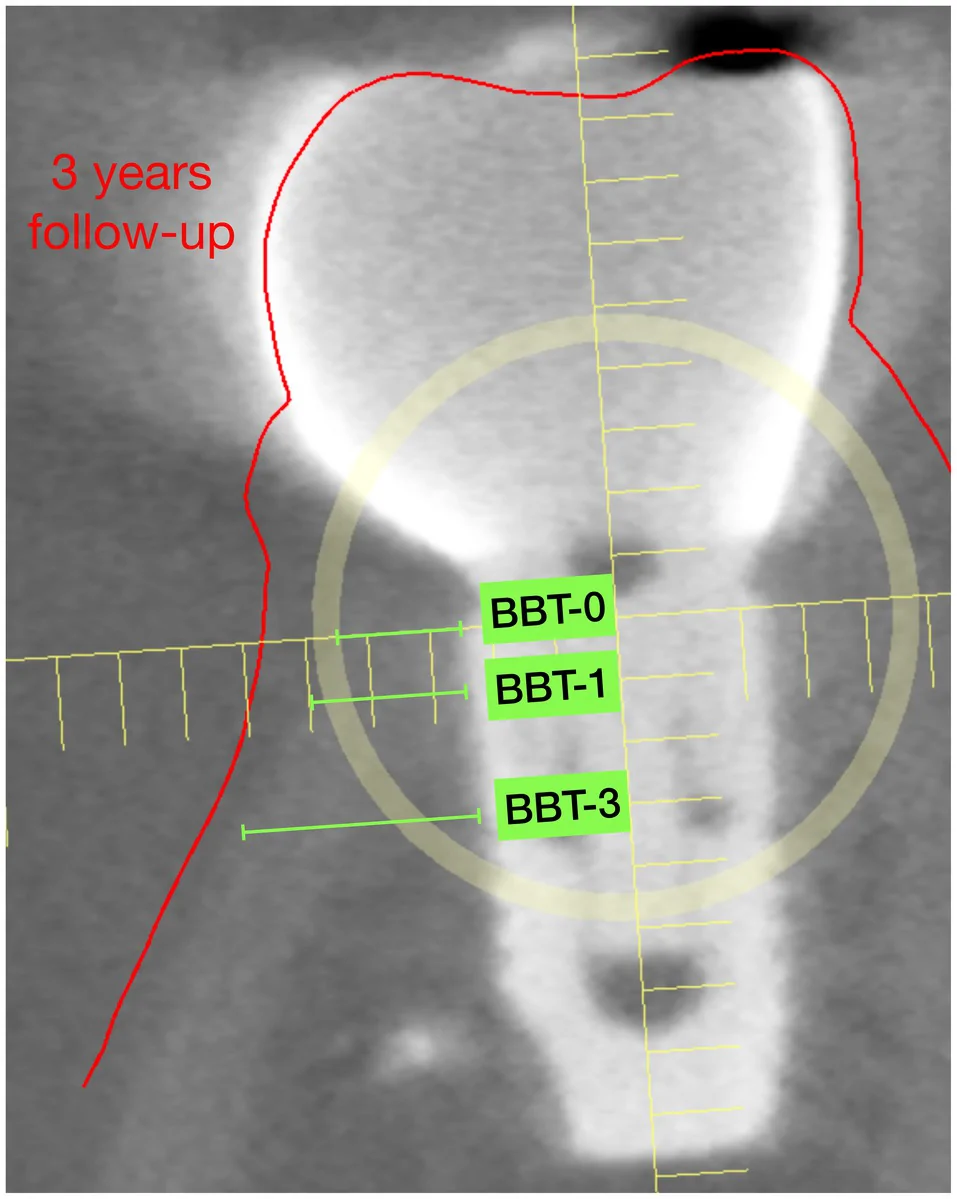
Schematic illustration of buccal bone thickness (BBT) measurements. A bucco‐oral cross‐section perpendicular to the central implant axis was used for the analysis. BBT was measured perpendicular to the implant axis at the implant shoulder (BBT‐0) and at 1 mm (BBT‐1) and 3 mm (BBT‐3) apical to the implant shoulder.

### 2.6 Secondary Outcome Measures

#### 2.6.1 Peri‐Implant Clinical Parameters

At 3 years after implant loading, the following clinical and peri‐implant parameters were recorded at six sites per implant (mesio‐buccal, mid‐buccal, disto‐buccal, mesio‐oral, mid‐oral, and disto‐oral) using a calibrated periodontal probe (PCPUNC 15, Hu‐Friedy, Chicago, IL, USA): probing depth (PD), measured from the mucosal margin to the bottom of the sulcus; bleeding on probing (BOP), recorded as present or absent within 15 s after gentle probing; plaque index (PCR), documented dichotomously as the presence or absence of visible plaque and the width of keratinized mucosa (KM), measured mid‐facially from the mucosal margin to the mucogingival junction. All measurements were rounded to the nearest 0.5 mm and performed by a calibrated examiner under standardized clinical conditions.

#### 2.6.2 Buccal Bone Thickness

In order to assess buccal bone thickness (BBT) at 3 years after implant loading, cone‐beam computed tomographic (CBCT) scans and digital surface models (STL files) were analyzed. The DICOM datasets from the CBCT scans and the corresponding STL surface scans derived from optical impressions were imported into a digital imaging software (SMOP, Swissmeda AG, Zurich, Switzerland) and superimposed using a best‐fit algorithm. In the bucco–oral cross‐section (region of interest), BBT was measured perpendicular to the implant surface at three levels: implant shoulder, 1 and 3 mm below the implant shoulder.

#### 2.6.3 Marginal Bone Loss

Marginal bone levels (MBL) were assessed at the 3‐year follow‐up by measuring the distance between the implant shoulder and the first bone‐to‐implant contact on both mesial and distal aspects. The thread pitch distance of the implant was used to calibrate all apico‐coronal measurements in each radiograph, which were analyzed using open‐source image analysis software (ImageJ, National Institutes of Health, Bethesda, MD, USA).

#### 2.6.4 Patient‐Reported Outcome Measures (PROMs)

At the 3‐year follow‐up, all patients completed a standardized questionnaire (Oral Health Impact Profile‐14; OHIP‐14) to assess patient‐reported outcomes.

### 2.7 Statistical Analysis

The statistical analysis followed that of the initial publication [12]. Given the limited sample size, analyses were descriptive and exploratory. Descriptive statistics (mean, SD, median, Q1, Q3) were calculated for all continuous variables. Statistical analyses were performed using GraphPad Prism v9 (GraphPad Software, San Diego, CA, USA) and SPSS v27.0 (IBM Corp., Armonk, NY, USA).

## 3 Results

### 3.1 Study Population

Baseline participant characteristics are presented in Table 1. The CONSORT flow diagram is shown in Figure 2 and representative cases from each study group, together with the corresponding measurements, are presented in Figure 3. During the follow‐up period from crown delivery to the 3‐year follow up examination, two of the 18 patients analyzed at 1 year were not available for the 3‐year assessment. One patient in the SCTG group received treatment for peri‐implantitis and was excluded from the 3‐year analysis because the intervention altered the peri‐implant tissue conditions under evaluation. One patient in the negative control group was lost to follow‐up after moving to a nursing home and expressed the wish to discontinue participation. The remaining 16 participants completed the 3‐year follow‐up and were included in the final analysis. The distribution of patients at 3 years of follow‐up was as follows: SCTG (*n* = 5), GBR (*n* = 6), and control (*n* = 5). The intra‐examiner reliability of marginal bone level (MBL) and buccal bone thickness (BBT) was calculated using the intra‐class correlation coefficients (ICC).

**TABLE 1:** Participants baseline characteristics.

	SCTG	GBR	Control
*N*	5	6	5
Age, mean (SD)	58.4 (5.5)	58.7 (14.3)	56.6 (19.9)
Gender			
Female	4 (80%)	1 (17%)	2 (40%)
Male	1 (20%)	5 (83%)	3 (60%)
Implant location			
Maxilla, *n* (%)	1 (20%)	4 (67%)	1 (20%)
Mandible, *n* (%)	4 (80%)	2 (33%)	4 (80%)
Implant position			
Premolar, *n* (%)	3 (60%)	5 (83%)	2 (40%)
Molar, *n* (%)	2 (40%)	1 (17%)	3 (60%)
Defect depth at implant placement (mm), median (IQR)	3.0 (1.8–3.0)	2.8 (1.5–3.0)	n/a
Width of keratinized mucosa (mm), median (IQR)	3.0 (2.0–5.0)	2.0 (1.0–3.0)	3.0 (2.0–3.5)
Probing depth (mm), mean (SD)	1.8 (0.3)	2.1 (0.7)	2.2 (0.4)

*Note:* Values are presented as mean ± standard deviation (SD) or median (interquartile range).

Abbreviations: GBR, guided bone regeneration; IQR, interquartile range; n/a, not applicable (control implants without buccal dehiscence); SCTG, subepithelial connective tissue graft.

**FIGURE 2:**
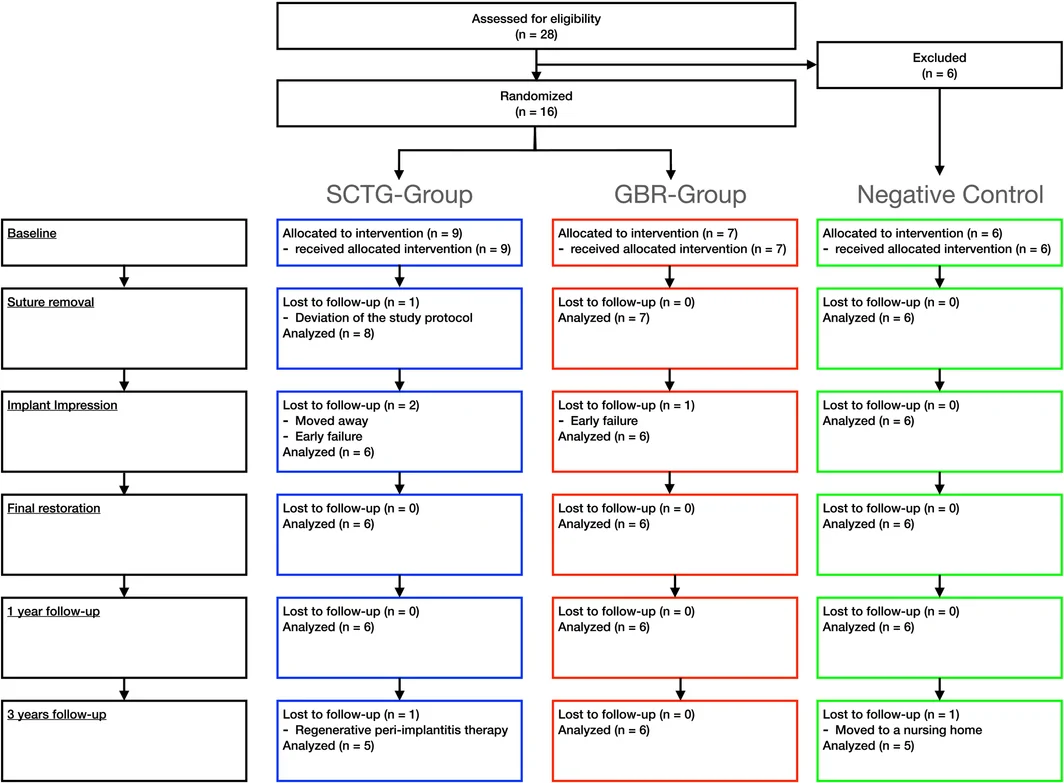
Flow diagram.

**FIGURE 3:**
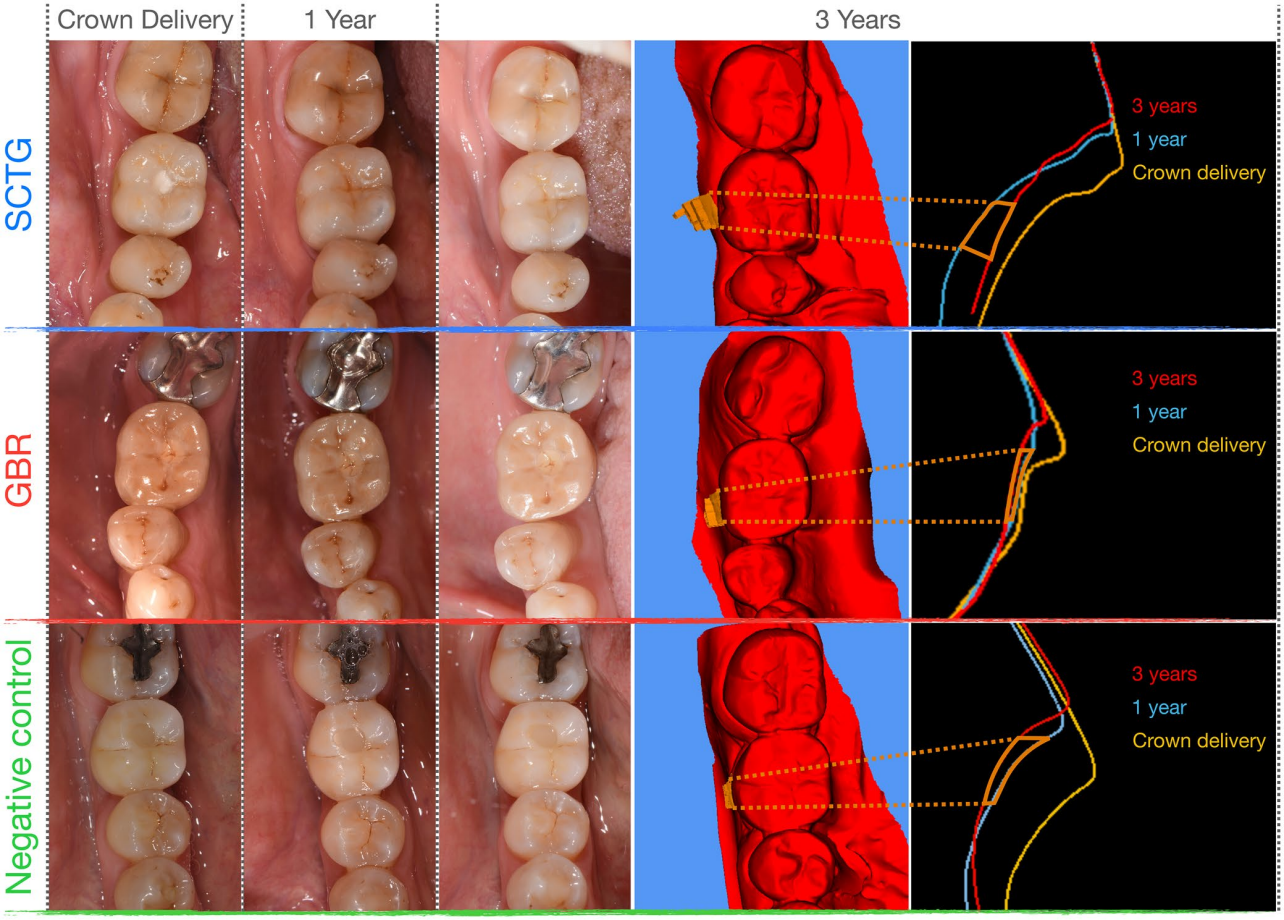
Clinical and volumetric changes in the peri‐implant soft‐tissue contour from crown delivery (baseline) to the 3‐year follow‐up, assessed at 1 mm and 3 mm apical to the mid‐facial mucosal margin. GBR, guided bone regeneration; NC, negative control; SCTG, subepithelial connective tissue graft.

The negative control group consisted of patients without buccal bone dehiscences at implant placement and served as a non‐randomized descriptive reference cohort. It was not included in the randomized comparison between the SCTG and GBR groups. Accordingly, comparisons involving the control group are descriptive only and should not be interpreted as inferential. Longitudinal analyses included assessments performed at baseline, 1, and 3 years.

### 3.2 Buccal Contour Changes

Profilometric contour changes between the 1‐ and 3‐year follow‐up examinations are summarized in Table 2 and illustrated in Figure 4. Representative clinical photographs and corresponding profilometric analyses for each treatment group are shown in Figure 3. At 1 mm below the mucosal margin, the median buccal contour change was 0.19 mm (Q1: −0.28; Q3: 0.29) in the SCTG group, 0.11 mm (Q1: −0.20; Q3: 0.30) in the GBR group, and 0.27 mm (Q1: −0.03; Q3: 0.35) in the negative control group. At 3 mm below the mucosal margin, the corresponding median contour changes were 0.05 mm (Q1: −0.36; Q3: 0.31) for SCTG, −0.05 mm (Q1: −0.22; Q3: 0.07) for GBR, and 0.11 mm (Q1: −0.11; Q3: 0.18) for the negative control group.

**TABLE 2:** Profilometric contour changes over time at the different regions of interest (ROI).

Variable	*n*	SCTG		*n*	GBR		*n*	Negative control	
ROI		Mean (SD)	Median (Q1, Q3)		Mean (SD)	Median (Q1, Q3)		Mean (SD)	Median (Q1, Q3)
ROI‐1									
∆Baseline—1 year	5	0.55 (0.64)	0.83 (−0.10; 1.07)	6	−0.03 (0.52)	−0.29 (−0.42; 0.50)	5	0.19 (0.44)	0.07 (−0.17; 0.63)
∆1 year—3 years	5	0.04 (0.31)	0.19 (−0.28; 0.29)	6	0.05 (0.34)	0.11 (−0.20; 0.30)	5	0.18 (0.20)	0.27 (−0.03; 0.35)
ROI‐3									
∆Baseline—1 year	5	0.67 (0.70)	0.97 (0.12, 1.08)	6	−0.16 (0.41)	−0.15 (−0.61, 0.29)	5	0.04 (0.34)	−0.07 (−0.24, 0.37)
∆1 year—3 years	5	−0.01 (0.37)	0.05 (−0.36, 0.31)	6	−0.06 (0.17)	−0.05 (−0.22, 0.07)	5	0.05 (0.15)	0.11 (−0.11, 0.18)

Abbreviations: GBR, guided bone regeneration; Q1, Q3, quartile 1, 3; ROI, region of interest; SCTG, subepithelial connective tissue graft; SD, standard deviation.

**FIGURE 4:**
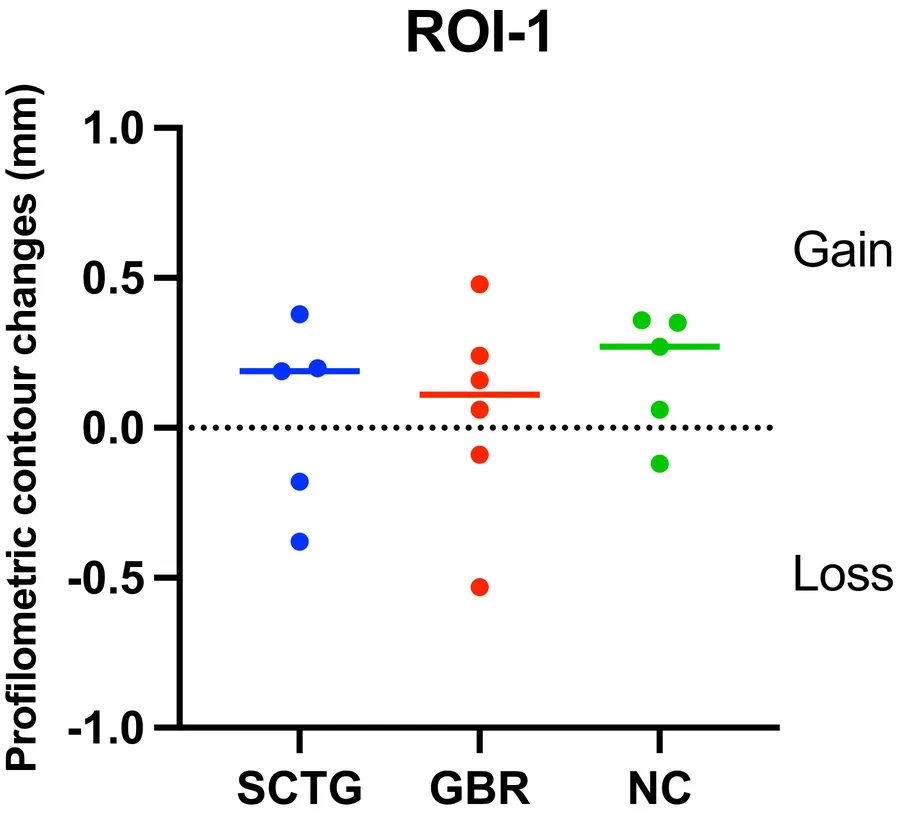
Profilometric contour changes at the 3‐year follow‐up at region of interest 1 (ROI‐1), located 1 mm apical to the mid‐facial mucosal margin. GBR, guided bone regeneration; NC, negative control; SCTG, subepithelial connective tissue graft.

Linear changes in the horizontal width of peri‐implant tissues between 1 and 3 years follow‐up are summarized in Table 3 and illustrated in Figure 1. At 1 mm below the midfacial mucosal margin, the median peri‐implant tissue width change was −0.32 mm (Q1: −0.62; Q3: 0.33) in the SCTG group, −0.17 mm (Q1: −0.53; Q3: 0.15) in the GBR group, and 0.10 mm (Q1: −0.23; Q3: 0.32) in the negative control group. At 3 mm below the mucosal margin, the corresponding median tissue width changes were −0.14 mm (Q1: −0.86; Q3: 0.19) for SCTG, −0.03 mm (Q1: −0.37; Q3: 0.09) for GBR, and −0.18 mm (Q1: −0.32; Q3: 0.33) for the negative control group.

**TABLE 3:** Changes in the horizontal width of peri‐implant tissue over time.

Variable	*n*	SCTG		*n*	GBR		*n*	Negative control	
		Mean (SD)	Median (Q1, Q3)		Mean (SD)	Median (Q1, Q3)		Mean (SD)	Median (Q1, Q3)
TW‐1									
∆Baseline—1 year	5	0.73 (0.81)	0.97 (−0.08, 1.42)	6	−0.33 (0.72)	−0.15 (−0.78, 0.23)	5	0.04 (0.61)	0.07 (−0.54, 0.63)
∆1 year—3 years	5	−0.18 (0.52)	−0.32 (−0.62, 0.33)	6	−0.20 (0.37)	−0.17 (−0.53, 0.15)	5	0.05 (0.31)	0.10 (−0.23, 0.32)
TW‐3									
∆Baseline—1 year	5	0.70 (0.86)	1.05 (0.00, 1.24)	6	−0.36 (0.81)	−0.03 (−0.71, 0.04)	5	−0.18 (0.39)	−0.25 (−0.52, 0.19)
∆1 year—3 years	5	−0.29 (0.54)	−0.14 (−0.86, 0.19)	6	−0.16 (0.39)	−0.03 (−0.37, 0.09)	5	−0.03 (0.35)	−0.18 (−0.32, 0.33)

Abbreviations: GBR, guided bone regeneration; Q1, Q3, quartile 1, 3; SCTG, subepithelial connective tissue graft; SD, standard deviation; TW1, Tissue Width at 1 mm below the mucosal margin; TW3, Tissue Width at 3 mm below the mucosal margin.

### 3.3 Peri‐Implant Parameters

Peri‐implant parameters are presented in Table 4. At the 3‐year follow‐up, the mean probing depth (PD) was 3.4 mm in the SCTG group, 3.1 mm in the GBR group, and 3.1 mm in the negative control group. Plaque control record (PCR) and bleeding on probing (BOP) values ranged between 10% and 30%, indicating overall healthy peri‐implant conditions across all groups. The mean midfacial width of keratinized mucosa (KM) was 3.0 mm in the SCTG group, 2.1 mm in the GBR group, and 3.0 mm in the control group. One implant in the SCTG group was diagnosed with peri‐implantitis during the observation period and was treated by regenerative surgical therapy before the 3‐year examination. This site was therefore not available for the 3‐year analysis.

**TABLE 4:** Clinical parameters at 3‐year follow‐up.

Variable	*n*	SCTG		*n*	GBR		*n*	Negative control	
		Mean (SD)	Median (Q1, Q3)		Mean (SD)	Median (Q1, Q3)		Mean (SD)	Median (Q1, Q3)
PD	5	3.4 (1.1)	2.8 (2.4, 4.6)	6	3.1 (0.7)	2.9 (2.6, 3.8)	5	3.1 (0.7)	3.5 (2.3, 3.7)
BOP	5	0.3 (0.2)	0.3 (0.0, 0.6)	6	0.3 (0.2)	0.3 (0.1, 0.6)	5	0.1 (0.2)	0.1 (0.0, 0.3)
PCR	5	0.3 (0.2)	0.3 (0.0, 0.5)	6	0.2 (0.1)	0.3 (0.1, 0.5)	5	0.1 (0.2)	0.0 (0.0, 0.3)
Keratinized mucosa width (mm)	5	3.0 (2.0)	4.0 (1.0, 4.5)	6	2.1 (1.2)	2.0 (1.0, 3.0)	5	3.0 (1.4)	3.0 (2.0, 4.0)

Abbreviations: BOP, bleeding on probing; GBR, guided bone regeneration; PCR, plaque control record; PD, probing depth; Q1, Q3, quartile 1, 3; SCTG, subepithelial connective tissue graft; SD, standard deviation.

### 3.4 Buccal Bone Thickness

The median buccal bone thickness (BBT) values at 1 mm (BBT‐1) and 3 mm (BBT‐3) below the implant shoulder are presented in Table 5. At the 3‐year follow‐up (Figure 1), the median peri‐implant buccal bone thickness at the level of the implant shoulder (BBT‐0) was 0.05 mm (Q1: 0.00; Q3: 2.06) in the SCTG group, 0.55 mm (Q1: 0.00; Q3: 2.73) in the GBR group, and 1.34 mm (Q1: 0.35; Q3: 2.32) in the control group. The intra‐examiner reliability for buccal bone thickness measurements was excellent, with an intraclass correlation coefficient (ICC) of 0.96 (95% CI: 0.79–0.99).

**TABLE 5:** Peri‐implant bone level (MBL) and the buccal bone thickness (BBT) of the different groups at 3 years follow‐up.

Variable	*n*	SCTG		*n*	GBR		*n*	Negative control	
		Mean (SD)	Median (Q1, Q3)		Mean (SD)	Median (Q1, Q3)		Mean (SD)	Median (Q1, Q3)
MBL	5	0.05 (0.2)	0.0 (−0.1, 0.2)	6	1.0 (1.3)	0.3 (0.0, 2.1)	5	0.3 (0.5)	0.2 (−0.1, 0.9)
BBT									
BBT‐0	5	0.83 (1.50)	0.05 (0.00, 2.06)	6	1.25 (1.37)	0.55 (0.00, 2.73)	5	1.34 (1.04)	1.34 (0.35, 2.32)
BBT‐1	5	1.47 (1.63)	1.53 (0.02, 2.90)	6	1.36 (1.45)	0.82 (0.00, 2.26)	5	1.71 (1.02)	1.74 (0.80, 2.60)
BBT‐3	5	2.02 (1.72)	2.26 (0.46, 3.46)	6	1.51 (1.56)	0.98 (0.64, 2.62)	5	2.33 (1.34)	2.07 (1.20, 3.73)

Abbreviations: BBT, buccal bone thickness measured at the implant shoulder (BBT‐0) and at 1 mm (BBT‐1) and 3 mm (BBT‐3) apical to the implant shoulder; GBR, guided bone regeneration; Q1, Q3, quartile 1, 3; SCTG, subepithelial connective tissue graft; SD, standard deviation; T, time points.

### 3.5 Marginal Bone Loss

At the 3‐year follow‐up, the median MBL measured 0.0 mm (Q1: −0.1, Q3: 0.2) in the SCTG group, 0.3 mm (Q1: 0.0, Q3: 2.1) in the GBR group, and 0.2 mm (Q1: −0.1, Q3: 0.9) in the control group (Table 5). The intra‐examiner reliability for MBL measurements was excellent, with an intraclass correlation coefficient (ICC) of 0.99 (95% CI: 0.96–0.99).

### 3.6 Patient‐Reported Outcome Measures (PROMs)

Based on the OHIP‐14 questionnaire, oral health‐related quality of life remained high across all groups (Table 6). At the 3‐year follow‐up, the median OHIP‐14 scores were 0.0 (Q1: 0.0; Q3: 1.0) in the SCTG group, 0.0 (Q1: 0.0; Q3: 3.0) in the GBR group, and 3.0 (Q1: 0.0; Q3: 8.0).

**TABLE 6:** Patient‐reported outcome measures (PROMs) at 3 years follow‐up.

	*n*	SCTG		*n*	GBR		*n*	Negative control	
		Mean (SD)	Median (Q1, Q3)		Mean (SD)	Median (*Q*1, *Q*3)		Mean (SD)	Median (Q1, Q3)
OHIP‐14	5	0.4 (0.9)	0.0 (0.0, 1.0)	6	1.7 (3.4)	0.0 (0.0, 3.0)	5	3.8 (4.2)	3.0 (0.0, 8.0)

Abbreviations: GBR, guided bone regeneration; OHIP‐14, Oral Health Impact Profile‐14; Q1, Q3, quartile 1, 3; SCTG, subepithelial connective tissue graft; SD, standard deviation; T, time points.

## 4 Discussion

The present 3‐year pilot randomized controlled trial comparing SCTG and GBR for the treatment of small buccal dehiscence defects (≤ 3 mm) predominantly revealed that: (1) both augmentation techniques maintained a stable buccal contour over 3 years with minimal volumetric changes, (2) peri‐implant clinical parameters remained stable, accompanied with favorable patient‐reported outcomes and (3) marginal bone levels and buccal bone thickness were preserved throughout the observation period, with no evidence of progressive bone loss.

Buccal contour stability remained largely unchanged between 1 and 3 years across all treatment groups, confirming that most tissue remodeling occurs during the early healing and prosthetic phases. This observation corroborates previous findings demonstrating that the majority of contour changes occur within the first year following restoration [4]. Sites augmented with SCTG exhibited long lasting stability beyond the first year. During the initial year, SCTG‐treated sites exhibited greater stability than those treated with GBR [12]. This finding supports the concept that soft‐tissue augmentation directly increases mucosal volume and promotes long‐term contour stability, rather than relying on secondary soft‐tissue adaptation over regenerated bone [12, 13, 21].

However, the favorable contour outcomes observed following SCTG should not be interpreted as evidence that hard‐tissue augmentation is unnecessary. Buccal bone has been proposed to play an important role in maintaining peri‐implant tissue health, and previous studies have suggested that inadequate buccal bone thickness may increase susceptibility to peri‐implant disease [10]. Although the present study demonstrated stable clinical parameters, preserved marginal bone levels, and favorable soft‐tissue contours over a 3‐year period, it was not designed to determine whether leaving buccal dehiscence defects untreated influences the long‐term risk of peri‐implantitis. Therefore, the biological implications of this treatment approach remain uncertain. It can only be hypothesized that improved soft‐tissue thickness and coverage may provide a more favorable peri‐implant environment by reducing exposure of the implant surface and improving tissue resilience. Nevertheless, this hypothesis remains unproven. In fact, the EFP S3 Clinical Practice Guideline concluded that there is currently no evidence that soft‐tissue augmentation alone in sites with thin peri‐implant tissues (< 2 mm) reduces the incidence of peri‐implant diseases [22]. Therefore, while SCTG appears to be an effective strategy for maintaining peri‐implant soft‐tissue contours in small buccal dehiscence defects, longer‐term studies are required to determine whether this approach provides biological benefits comparable to those achieved with hard‐tissue augmentation.

It is noteworthy that sites treated with GBR remained stable between 1 and 3 years of follow‐up, whereas the greatest dimensional changes occurred during the first year. These early changes are likely related to the nature of the GBR procedure itself, as clinicians often intentionally over‐augment to compensate for the expected graft resorption during healing [23]. Another possible explanation for the early changes is the influence of individual bone phenotype [24]. Theoretically, any augmentation that exceeds the patient's phenotypic boundary is prone to resorption, leading the alveolar ridge to return toward its original, phenotype‐determined dimension [24]. In this context, soft‐tissue augmentation may offer an advantage over GBR, as the mucosal phenotype does not impose a comparable dimensional limitation. By directly increasing tissue thickness, soft‐tissue grafts may provide a more stable and phenotype‐adaptive contour from the outset, without the risk of substantial graft resorption. However, this hypothesis remains speculative as mentioned and needs to be proven.

Implant diameter selection may also reduce the likelihood of buccal dehiscence formation, as narrower implants better conform to the available buccal bone envelope. In the present study, standard‐diameter implants (3.6–4.2 mm) were placed in a prosthetically driven position, and narrower implants may have prevented some dehiscence defects. This consideration should be incorporated into clinical decision‐making when planning implant placement in the posterior region.

One implant in the SCTG group developed peri‐implantitis and was excluded from the 3‐year assessment. All groups otherwise exhibited healthy peri‐implant conditions characterized by low probing depths, minimal bleeding on probing, and low plaque scores, with no clinically relevant differences between SCTG and GBR. These findings indicate that both augmentation strategies support stable peri‐implant health when performed under proper maintenance conditions and with appropriate prosthetic design. The mean width of KM was slightly greater in the SCTG group (3.0 mm) compared with the GBR group (2.0 mm), consistent with previous reports demonstrating that connective‐tissue grafting enhances soft‐tissue dimensions [4, 7]. Despite these soft‐tissue differences, peri‐implant parameters related to inflammation (PD, BOP, PCR) were comparable across groups, confirming that the type of augmentation procedure does not influence peri‐implant tissue health in the mid‐term [25].

Radiographically, marginal bone levels remained stable between 1 and 3 years, with no evidence of progressive bone loss in any treatment group. These results confirm that implants placed in sites with small buccal dehiscences can achieve and maintain bone stability when defects are augmented and patients adhere to regular maintenance care [11]. These observations are in line with recent evidence highlighting the protective role of peri‐implant mucosal thickness in maintaining contour and marginal bone stability over time [4, 7, 20].

As for patient‐reported outcomes, neither treatment had a major impact on oral health–related quality of life, as assessed using the OHIP‐14 questionnaire. This finding was not unexpected, given that the difference between the two procedures was purely surgical and that a certain degree of recall bias is inherent to a 3‐year follow‐up. Moreover, it has been suggested that the OHIP‐14 focuses primarily on psychological aspects and may lack the sensitivity to detect subtle differences in patients undergoing single implant placement [26].

The present study has several limitations that should be considered when interpreting the results. First, this was a pilot study with a small sample size and an exploratory design. Consequently, no inferential statistical analyses were performed and the study was not powered to detect differences between groups. The results should therefore be interpreted descriptively and regarded as hypothesis‐generating rather than confirmatory. Furthermore, the per‐protocol analysis may have introduced attrition bias, as patients lost to follow‐up could have differed systematically from those who completed the study. Second, CBCT and profilometric analyses are inherently subject to registration inaccuracies and limited spatial resolution, particularly when assessing thin buccal bone adjacent to titanium implants. Consequently, the reported buccal bone thickness values represent radiographically detectable bone [27] rather than the true histological bone thickness. This limitation is especially relevant for SCTG‐treated sites, where buccal bone regeneration was not expected because the procedure was designed exclusively for soft‐tissue augmentation. Third, the study population consisted of carefully selected and well‐maintained patients, which may limit the generalizability of the findings to routine clinical practice. Moreover, only one SCTG protocol and one GBR protocol were evaluated. Fourth, the control group was not randomized and should therefore be regarded solely as a descriptive reference cohort. In addition, detailed baseline variables such as soft‐tissue thickness and defect morphology (contained versus non‐contained) were not systematically recorded, which may have influenced healing and limits interpretation of the augmentation outcomes. Finally, although the present study provides one of the longest follow‐ups currently available for this clinical scenario, 3 years still represents a mid‐term evaluation. Longer‐term studies are required to determine whether the favorable clinical and volumetric outcomes observed after SCTG and GBR are maintained over time.

## 5 Conclusions

Within the limitations of this pilot study, both SCTG and GBR were associated with stable buccal contour conditions and favorable peri‐implant health over a 3‐year follow‐up period. These findings were observed under adequate self‐performed plaque control and adherence to supportive care. Adequately powered RCTs are needed to confirm these findings.

## Funding

The authors have nothing to report.

## Conflicts of Interest

The authors declare no conflicts of interest.

## Supporting information


**Data S1:** CONSORT 2025 checklist of information to include when reporting a randomized trial.

## Data Availability

The data that support the findings of this study are available from the corresponding author upon reasonable request.
